# A rare diaphragmatic ureteral herniation case report: endoscopic and open reconstructive management

**DOI:** 10.1186/s12894-017-0207-5

**Published:** 2017-04-05

**Authors:** Frank C. Lin, Jamie S. Lin, Samuel Kim, Jonathan R. Walker

**Affiliations:** 1 Division of Urology, Department of Surgery, University of Arizona, 1501 N. Campbell Ave, Tucson, AZ 85724 USA; 2 Renal-Electrolyte and Hypertension Division, Department of Medicine, University of Pennsylvania, Perelman School of Medicine, 3400 Civic Blvd, Philadelphia, PA 19104 USA; 3 Division of Cardiothoracic Surgery, Department of Surgery, University of Arizona, 1501 N. Campbell Ave, Tucson, AZ 85724 USA

**Keywords:** Ureter, Ureteral hernia, Diaphramatic hernia, Ureteral reconstruction, Renal obstruction, Case report

## Abstract

**Background:**

Ureteral herniations are a rare occurrence, generally found incidentally on cross sectional imaging or during surgical intervention for unrelated processes. Several locations of ureteral herniations can occur including the inguinal, femoral, sciatic, obturator, and thoracic regions. While few reports of ureteral hernias are reported in the literature overall, the vast majority of those reported are inguinoscrotal herniations found during evaluation and treatment of inguinal hernias. Pelvic outlet ureteral herniations intrinsically are more common secondary to their dependent locations. Intrathoracic ureteral herniations through diaphragmatic defects are an exceptionally rare subset of ureteral herniations and have only been described sparingly. Fewer than ten case reports of diaphramatic ureteral herniations have been reported and none have described both cystoscopic management and open reconstruction.

**Case presentation:**

We report the case of a 81 year old female with flank pain who was found to have idiopathic diaphragmatic hernia with incarcerated proximal ureter. She had no prior injury or surgery that explained her clinical presentation. She was initially observed and then managed conservatively with ureteral stent exchanges. Ultimately she underwent open surgical repair of her diaphragmatic hernia, reduction, resection and anastomosis of redundant proximal incarcerated ureteral segment, and nephropexy for a hypermobile right renal unit. This case report illustrates the pre- and post-operative imaging studies of a very rare intrathoracic ureteral herniation as well as surgical approach to repair.

**Conclusion:**

A herniated ureter is a potential source of serious renal and ureteral complications. The thoracic herniation of ureter is the rarest of the ureteral herniations. When discovered, they should be managed to preserve renal function and prevent strangulation of the affected segment of ureter. This case report documents the treatment of a thoracic ureteral herniation with observation, conservative endoscopic management, and finally open surgical reconstruction.

## Background

Ureteral herniations are a rare occurrence normally found incidentally on imaging or during surgical hernia correction and can be a cause of ureteral obstruction [1]. These herniations can occur in several locations including the inguinal, femoral, sciatic, obturator, and thoracic regions [2]. Diaphragmatic herniations, however, are the rarest form of ureteral herniations with fewer than ten cases reported since 1958 [3]. They have been identified in the retrocrural area [4] and through congenital Bochdalek hernias [5–7].

Prior cases of obstructing diaphragmatic ureteral hernias have been managed with ureteral stent placement or open reduction, but none have described situations requiring open hernia reduction and ureteral reconstruction [8]. Here, we present a unique case of a right-sided posterior diaphragmatic hernia containing an incarcerated right proximal ureter with subsequent hydroureteronephrosis, and what we believe to be the first documented open-reduction and reconstruction of a thoracic ureteral herniation.

## Case presentation

**Fig. 1:**
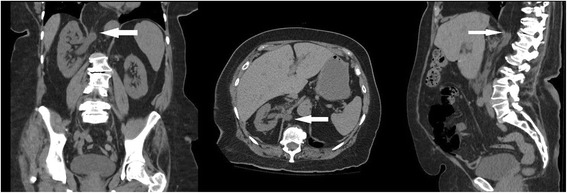
Patient’s initial computed tomography (CT) at presentation demonstrating small loop of ureter within right-sided diaphragmatic hernia. Coronal, Axial, Sagittal views with white arrow showing segment of herniated ureter

**Fig. 2:**
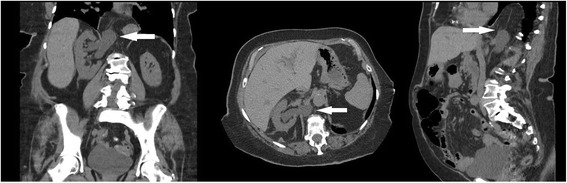
Follow up computed tomography (CT) 18 months later demonstrating increased hydroureteronephrosis and increased segment of entrapped ureter. Coronal, Axial, Sagittal views with white arrow showing segment of herniated ureter

The patient was an 81-year-old Caucasian woman with a history of persistent right-sided flank pain with no associated symptoms including dysuria, hematuria, frequency, or urinary retention. She had no history of prior abdominal surgery, trauma, or congenital defects. Given the unclear etiology, we obtained cross-sectional imaging which demonstrated a right-sided Bochdalek diaphragmatic hernia incarcerating her proximal ureteral segment (Fig. 1). Initially, she was managed conservatively with surveillance monitoring since her pain was tolerable and her renal function was preserved. Although her kidney function remained unchanged, follow-up imaging at the next visit surprisingly demonstrated an interval increase in hydroureteronephrosis (Fig. 2). Additionally, the entrapped ureteral portion had progressed with now obvious hydronephrosis of the renal pelvis. A Tc-99m MAG-3 nuclear medicine renal scan with furosemide confirmed our suspicion – she had moderate obstruction of the right kidney. This study also demonstrated an asymmetric split function with 64% left and 36% right in the setting of a stable baseline creatinine (0.7mg/dL).

In attempt to reduce the entrapped ureter and unobstruct the right kidney, a double-pigtail ureteral stent was endoscopically placed. We were able to reduce the herniation with the use of a super stiff wire and a standard soft stent was placed in the usual retrograde fashion. During her stent exchanges, it became apparent that the herniated ureteral segment was enlarging as the curl of the double-J stent retracted into the distal ureter requiring ureteroscopy to visualize and engage. Longer stents were used to ensure that the distal curl remained in the bladder lumen despite recurrent herniation. Stiffer stents were also used however these maneuvers eventually were no longer able to straighten and reduce the ureter, leaving it entrapped in the thoracic cavity. After several stent exchanges were completed, the patient became symptomatic with ureteral stent discomfort including flank pain, urinary frequency and urgency. Due to concern that her diaphragmatic defect was enlarging, we discussed definitive surgical options with her; nephrectomy versus resection and anastomoses of her right ureter with diaphragmatic hernia repair. She ultimately opted to preserve her right kidney and proceed with open-resection reduction and repair.

We approached her open surgical repair through a standard supra-11 incision carried down through her flank muscles where we entered the retroperitoneal space. First, we freed her affected kidney from the surrounding tissue and then identified the incarcerated ureteral segment using our previously placed stent. The redundant ureter was mobilized out of the diaphragmatic defect and returned to the retroperitoneal abdominal cavity. We closed her diaphragmatic defect using interrupted 0 silk sutures, and then excised the redundant proximal ureteral segment where we spatulated and reapproximated the proximal and distal ends. Last, we replaced the patient’s ureteral stent and completed the anastomosis over a 7-French x 26 cm double-J ureteral stent. Due to the dissection of the renal hilum and perinephric tissue, the kidney appeared to have more mobility. To help maintain proper drainage and reduce tension on the anastomosis, we performed a nephropexy, securing the posterior aspect of the kidney to the flank muscles with a 0 silk. From there, we performed the standard two-layered incision closure.

**Fig. 3:**
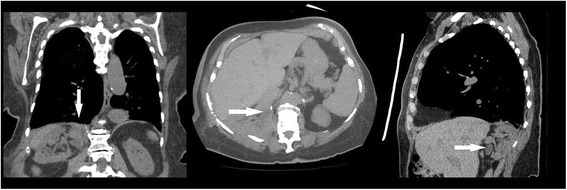
Post-surgical computed tomography (CT) demonstrating repair of diaphragmatic hernia and segmental resection of ureter. Coronal, Axial, Sagittal views with white arrow showing resolution of hydroureteronephrosis and repair of diaphragmatic hernia. Note the changes in the position of the right kidney from nephroxy and no redundant ureter is evident

The patient had an uncomplicated post-operative course and was discharged home after regaining bowel function and returning to her baseline physical activity on post-operative day three. There were no complications during surgery or recovery. Two months later, we removed her ureteral stent and obtained follow-up imaging which demonstrated repair of the diaphragmatic defect without hernia recurrence (Fig. 3). We also repeated the Tc-99m MAG-3 nuclear medicine renal scan, which now demonstrated normal clearance of the previously obstructed right kidney. Post operative creatinine was 0.7 mg/dL with no obstruction detected. Split cortical function was 54% left and 46% right; an improvement the patient’s preoperative findings.

## Discussion

Ureteral herniation is a rare anatomic entity. In 1975, Pollack et al. reported that there had been 120 reports of ureteral hernias at the time of their case series publication [9]. While the exact case number is unknown, recent publications have documented fewer than 200 cases [10, 11]. These herniations, have been described in several anatomic regions including inguinal, femoral, sciatic, obturator, and thoracic regions. Out of these listed, inguinal ureteral hernias are the most common, occurring approximately 42–64% of the time, and have the greatest risk of inadvertent injury due to the herniorrhaphies associated with that area [9, 12]. Conversely, literature review revealed fewer than 10 documented thoracic ureteral herniation cases and none were from sequelae to iatrogenic injuries. This subset likely represents the rarest form of ureteral herniations.

Thoracic ureteral hernias were first documented by Swithinbank in 1958 [3]. This case involved a right-sided diaphragmatic ureteral herniation in a 60 year-old female who was found to be symptomatic with right-sided flank pain. She ultimately underwent open-reduction of the ureteral hernia with repair of diaphragmatic defect; however, no reconstruction was performed and the redundant ureter was left in the retroperitoneal space. The paucity of these occurrences is thought to be secondary to their non-dependent anatomic location. From case reports, elderly patients appear to be at highest risk. While the etiology of this is unknown, this could largely be attributed to study bias in that cross sectional imaging is more frequently performed in the elderly population. Further metaanalysis of prior case reports and series may help elucidate a more clear pattern. Previous reports have also discussed thoracic variants, but ours is the first to detail open-reduction and reconstruction after progression of herniation and obstruction.

In general, ureteral hernias are an incidental finding. If symptomatic, they can present with flank pain, gross hematuria, renal dysfunction, nephrolithiasis, and urosepsis [11, 13]. While these symptoms are non-specific, and the likelihood of diagnosing a herniated ureter is low, the consequences of misdiagnosis can result in permanent ureteral injury, loss of renal function, and/or urosepsis. Diagnostic studies such as computer tomography, magnetic resonance imaging, intravenous and retrograde pyelography can be helpful in diagnosing this entity.

Ureteral hernias are managed conservatively unless there is an urgency to treat (e.g. obstruction, urosepsis, or intractable flank pain). These patients can generally be followed in clinic with both labs and imaging to monitor for progression. Isolated lab tests may be inadequate in detecting obstruction in the setting of a normal functioning contralateral kidney, as it may not reflect true kidney function as our patient demonstrated. If intervention is warranted, endoscopic stent placement is the routine management therapy. However, if endoscopy is unsuccessful in reducing the herniation, then open-reduction surgery may be a viable option for renal preservation, as we demonstrated. Care should be taken to avoid injuring the ureter with the end-goal of relieving the ureteric obstruction and ensuring that impairment of renal function is minimized. Each case should be evaluated on its own merit and follow-up imaging and diagnostics studies should be obtained to ensure successful reduction.

## Conclusion

Thoracic ureteral herniations are extremely uncommon, and imaging and functional studies are necessary for monitoring possible progression of the anatomic defect. Here, we demonstrate for the first time, a successful open-reduction and reconstructive surgery for an elderly women found to have an idiopathic thoracic ureteral herniation. We suggest that this approach may be an alternative option for patients that cannot be managed with routine endoscopic management.
